# forqs: forward-in-time simulation of recombination, quantitative traits and selection

**DOI:** 10.1093/bioinformatics/btt712

**Published:** 2013-12-10

**Authors:** Darren Kessner, John Novembre

**Affiliations:** ^1^Bioinformatics Interdepartmental Program, University of California, Los Angeles, CA 90095 and ^2^Department of Human Genetics, University of Chicago, Chicago, IL 60637, USA

## Abstract

**Summary:** forqs is a forward-in-time simulation of recombination, quantitative traits and selection. It was designed to investigate haplotype patterns resulting from scenarios where substantial evolutionary change has taken place in a small number of generations due to recombination and/or selection on polygenic quantitative traits.

**Availability and implementation**: forqs is implemented as a command-line C++ program. Source code and binary executables for Linux, OSX and Windows are freely available under a permissive BSD license: https://bitbucket.org/dkessner/forqs.

**Contact:** jnovembre@uchicago.edu

**Supplementary information:** Supplementary data are available at *Bioinformatics* online.

## 1 INTRODUCTION

Simulations have a long history in population genetics, both for verifying analytical results and for exploring population models that are mathematically intractable. Population genetics simulations can be broadly classified as forward-in-time (e.g. Wright–Fisher) or backward-in-time (e.g. coalescent). Coalescent simulations [e.g. ms (Hudson, 2002), MaCS (Chen *et al.*, 2009), fastsimcoal (Excoffier and Foll, 2011)] are efficient for simulating neutral sequence data because they only need to track lineages that are ancestral to the sample. Although it is possible to simulate certain selection scenarios within the coalescent framework (Ewing and Hermisson, 2010; Hudson and Kaplan, 1988), one must turn to forward-in-time simulations to model selection in a flexible way.

Many forward-in-time simulators are currently available. Most of these simulators use a mutation-centric approach, implemented by storing the mutations carried by individuals in an array. To handle selection, the majority of these simulators assign selection coefficients to individual mutations [e.g. ForwSim (Padhukasahasram *et al.*, 2008), Fregene (Chadeau–Hyam *et al.*, 2008), GENOMEPOP (Carvajal–Rodriguez, 2008), SFS_CODE (Hernandez, 2008), TreesimJ (O’Fallon, 2010), SLiM (Messer, 2013)], although a few also include support for quantitative traits [e.g. ForSim (Lambert *et al.*, 2008), quantiNemo (Neuenschwander *et al.*, 2008), simuPOP (Peng and Kimmel, 2005)]. Hoban *et al.* (2011) and Yuan *et al.* (2012) are recent reviews providing a comprehensive comparison of these and other simulators.

In many scenarios of biological interest, substantial evolutionary change has taken place in a small number of generations due to recombination and/or selection on standing variation, rather than mutational input. For example, one may be interested in the genome-wide haplotype patterns that emerge from admixture between historically isolated populations (Wegmann *et al.*, 2011) or from artificial selection on a quantitative trait. Studying these haplotype patterns can be difficult with existing forward-in-time simulators because detailed information about the mosaic haplotype structure of individuals is not readily available, and must be inferred from the output sequences of the simulation and/or stored recombination event data. In addition, forward-in-time simulators that store entire sequences incur a severe trade-off between the size of the genomic regions and the size of the populations simulated.

Motivated by such examples, we have implemented a new forward-in-time simulation approach that, instead of tracking single-site variants, tracks individual haplotype chunks as they recombine over multiple generations. Further, we have designed the simulator for fast simulation of quantitative traits under selection. We have labeled this software forqs (Forward-in-time simulation of Recombination, Quantitative Traits and Selection). Similar approaches have been implemented recently by Haiminen *et al.* (2013) and by Aberer and Stamatakis (2013) for the simple selection models with per-mutation fitness effects.

The haplotype-based design allows for fast simulation of whole genomes, with efficient memory usage. For example, forqs can easily simulate two populations (size 10 000 each) selected for different optimal trait values, where individuals have human-sized genomes (23 chromosome pairs, 100 Mb each), taking ∼2 s/generation. For comparison, existing forward simulators are limited by the amount of sequence that can be stored in arrays in memory: for the aforementioned 20 000 individuals, 16 GB of memory would permit the storage of only 3.2 million base pairs of sequence per individual, which is an order of magnitude smaller than the smallest human chromosome. The forqs’ design also preserves information about the haplotype structure of individuals, which allows for immediate identification of genomic regions where individuals share identical-by-descent haplotype tracts.

Our simulator uses a modular architecture to allow the user to flexibly specify recombination maps, mutation rates, demographic models, quantitative traits and fitness functions. This modular approach facilitates simulation of complicated scenarios and investigation of the resulting haplotype patterns. forqs is currently under active development to support ongoing projects.

## 2 DESIGN AND IMPLEMENTATION

**Fig. 1.**
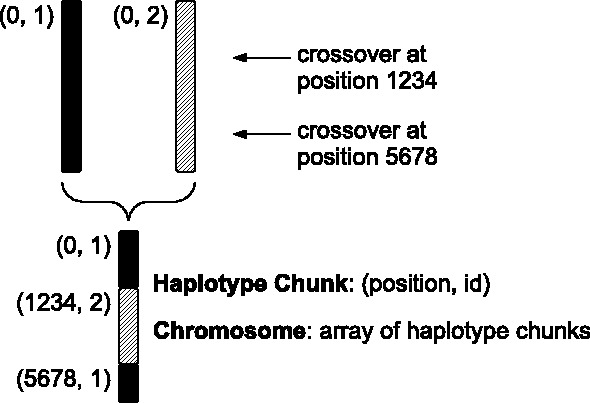
forqs chromosome representation. An individual chromosome is represented by a list of haplotype chunks. Each haplotype chunk is represented by two numbers *(position, id)*: the position where it begins and the identifier of the founding haplotype from which it is derived. This cartoon depicts a chromosome with three haplotype chunks as the result of recombination (double crossover) between two founder chromosomes

forqs begins with a set of founding haplotypes carried by the individuals in the initial generation. Individuals are diploid and carry a user-specified number of chromosome pairs. By assigning a unique identifier to each founding haplotype, individual haplotype chunks are tracked as they recombine over subsequent generations (Fig. 1). For the purposes of simulation, any existing neutral variation on the haplotype chunks can be ignored, and only those loci with fitness effects need to be tracked.

forqs performs the following actions during a single cycle of the simulation: (i) generation of new populations, (ii) genotyping, (iii) quantitative trait evaluation, (iv) fitness evaluation and (v) reporting. forqs has a flexible design in which the simulator delegates specific tasks or calculations to configurable modules. The user specifies which modules to instantiate in a configuration file.

In addition to the primary modules that are used to specify demography, mutation, recombination, quantitative traits, fitness and reported output, there are several building block modules that provide basic functionality to the primary modules. For example, Trajectory modules provide a unified method for specifying values that change over time, such as population sizes or migration rates. Similarly, Distribution modules can be used to specify how to draw particular random values [e.g. quantitative trait loci (QTL) positions or allele frequencies).

As an illustration of forqs configuration, suppose that a user wanted to simulate populations undergoing neutral admixture. The user would specify a PopulationConfigGenerator module representing a stepping stone or island model with the desired population size and migration rate trajectories. However, the user would not specify any quantitative trait modules and would use the default FitnessFunction module that assigns identical fitness values to all individuals. On the other hand, to simulate an artificial selection experiment with truncation selection on a single quantitative trait, the user would specify the trait with QTLs and effect sizes, and choose a FitnessFunction module that selects the desired proportion of individuals to produce the next generation. Alternatively, the user could indicate that the QTLs and effect sizes should be drawn randomly from user-specified distributions.

The representation of chromosomes as haplotype chunks in forqs makes efficient use of memory, independent of the size of the chromosomes. On a typical laptop computer, for a population size of 1 million, simulations take ∼1.5 s/generation for neutral simulations and ∼3 s/generation with selection at a single locus. Decreasing the population size allows the simulation of a greater number of generations in a reasonable amount of time: a population size of 10 000 takes ∼3 s/100 generations (without selection, with a slight increase with selection). However, forqs’ design comes with the trade-off that memory usage grows linearly with the number of generations simulated due to recombination. Thus, for investigations focusing on mutational input over a large number of generations (e.g. studies involving demographic changes taking place over thousands of generations), forqs’ design is not as efficient as array-based implementations (e.g. SLiM or SFS_CODE) that were designed specifically for these scenarios. Similarly, we recommend that forqs be used in conjunction with a coalescent simulator to generate neutral variation, rather than running forqs for a long burn-in period to reach mutation-drift equilibrium.

forqs has been extensively tested for correctness, both at the level of individual code units and in its large-scale behavior in comparison with theoretical predictions from population genetics and quantitative genetics. Validation results, tutorials and documentation can be found in the Supplementary Information. Configuration files for all simulations mentioned in this article are included in the forqs software packages.

## Supplementary Material

Supplementary Data
