# Supplementary material for: forqs: forward-in-time simulation of recombination, quantitative traits and selection
Source: Bioinformatics. 2013 Dec 10;30(4):576–7. doi: 10.1093/bioinformatics/btt712 (PMC3928523; doi:10.1093/bioinformatics/btt712)
Supplement: Supplementary Data [file supp_30_4_576__index.html]

forqs: Forward-in-time Simulation of Recombination, Quantitative Traits, and Selection — forqs: forward-in-time simulation of recombination, quantitative traits and selection — forqs: forward-in-time simulation of recombination, quantitative traits and selection — Supplementary Data 

# forqs: forward-in-time simulation of recombination, quantitative traits and selection

## Supplementary Data

files

**Files in this Data Supplement:**

- Supplementary Data - pdf file
