# Supplementary material for: forqs: forward-in-time simulation of recombination, quantitative traits and selection
Source: Bioinformatics. 2013 Dec 10;30(4):576–7. doi: 10.1093/bioinformatics/btt712 (PMC3928523; doi:10.1093/bioinformatics/btt712)
Supplement: Supplementary Data [file supp_btt712_forqs_suppinfo.pdf]

`forqs` Supplementary Information
Forward-in-time Simulation of Recombination, Quantitative Traits, and Selection

Darren Kessner and John Novembre

November 20, 2013

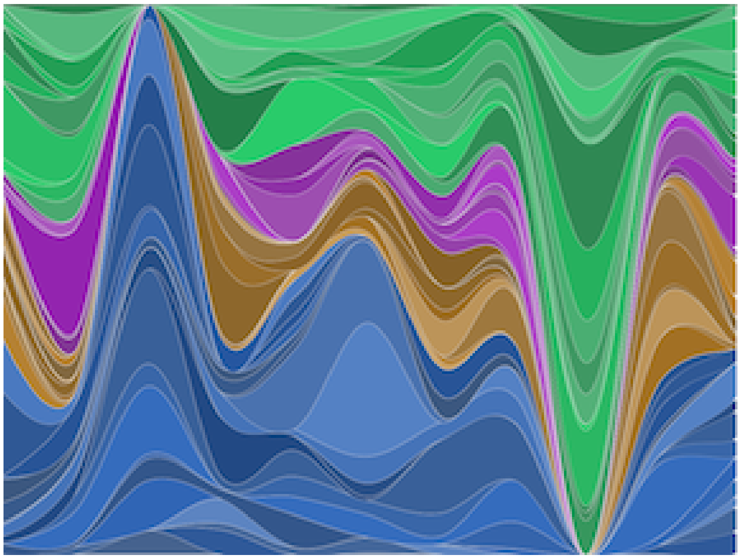

`forqs` is a forward-in-time simulation of recombination, quantitative traits, and selection. The `forqs` simulator was designed to investigate haplotype patterns resulting from scenarios where substantial evolutionary change has taken place in a small number of generations due to recombination and/or selection on polygenic quantitative traits. To do this, `forqs` tracks individual haplotype chunks during the course of the simulation; each chunk carries an identifier specifying the ancestral individual from which the block is derived. `forqs` is implemented as a command-line C++ program, using a modular design that gives the user great flexibility in creating custom simulations.

`forqs` is freely available with a permissive BSD license. Binary executables can be obtained for Linux, OSX, and Windows from:
`https://bitbucket.org/dkessner/forqs/downloads`

About the image

The image on the first page was created based on haplotype frequency information reported during a `forqs` simulation. The image shows local haplotype frequencies (equivalently, local ancestry proportions) along a single chromosome. A single population was simulated with selection at two loci (one near each end of the chromosome). 25% of individuals (blue) in the initial generation carried the selected allele at the first locus, and a different 25% (green) carried the selected allele at the second locus. After 100 generations, the two selected variants have fixed in the population. All individuals carry one of the blue haplotypes in the region containing the first locus, as well as one of the green haplotypes in the region containing the second locus.

Contents

1 Getting Started **4**

2 Design Overview **5**

3 Tutorial Introduction **7**
3.0 A minimal example 7
3.1 Recombination and reporting output 8
3.2 Wright-Fisher simulation 9
3.3 Selection 10
3.4 Trajectories 12
3.5 Quantitative traits 14
3.6 Reporting haplotype frequencies 16

4 Modules **19**
4.1 Module types and interfaces 19
4.2 Module specification and instantiation 19
4.3 Top-level module: SimulatorConfig 20
4.3.1 Global simulation parameters 20
4.3.2 References to primary modules 21
4.4 Primary modules 22
4.4.1 PopulationConfigGenerator 22
4.4.2 RecombinationPositionGenerator 23
4.4.3 MutationGenerator 23
4.4.4 VariantIndicator 24
4.4.5 QuantitativeTrait 24
4.4.6 FitnessFunction 24
4.4.7 Reporter 24
4.5 Building block modules 25
4.5.1 Locus and LocusList 25
4.5.2 Trajectory 25
4.5.3 Distribution 26

5 Simulating background variation **27**
5.1 Example 1: No new mutations 27
5.2 Example 2: Including new mutations 28

6 Validation **29**
6.1 Single locus selection 29
6.2 Decay of linkage disequilibrium 29
6.3 Mutation-drift equilibrium 30
6.4 Response to selection 31
6.5 Performance 32

7 Software development **34**
7.1 Building the program 34
7.2 Structure of the codebase 34
7.3 Software architecture 34
7.3.1 Low-level data structures 35
7.3.2 Configurable modules 35
7.3.3 Mutation handling 36
7.4 Appendix: Boost libraries 37
7.5 Appendix: Cross-compilation targeting Windows 37

1 Getting Started

forqs binaries (Linux, OSX, and Windows) are distributed in zip file packages containing the executables, documentation, and example configuration files. The latest forqs packages are available here:

https://bitbucket.org/dkessner/forqs/downloads

forqs is a command line program that takes a single argument specifying the configuration file for the simulation:

```
forqs config_file
```

The forqs packages include an `examples` directory containing several example configuration files. After unzipping the forqs package, you can run forqs directly from the package directory:

```
bin/forqs examples/example_1_locus_selection.txt
```

forqs puts all output files in the output directory specified in the configuration file. After running this command, you will find a new directory `output_example_1_locus_selection` with the output from this simulation. For convenience, you can put the forqs executable somewhere in your `PATH`, e.g. `~/bin`, so that you don't have to type the path when running the program.

In addition to the document you are now reading, forqs packages include the forqs Module Reference (`docs/forqs_module_reference.html`) which contains details about the configurable modules available to the user, including parameter names, usage, and links to examples.

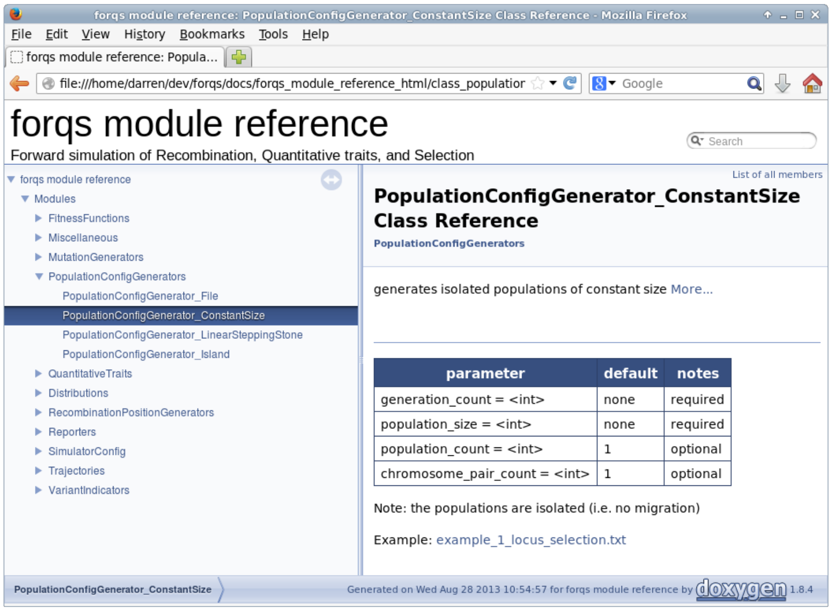

2 Design Overview

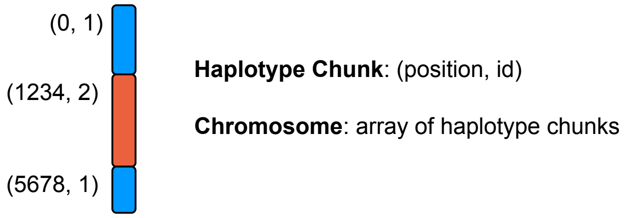

Figure 1: `forqs` **chromosome representation**. An individual chromosome is represented by a list of haplotype chunks. Each haplotype chunk is represented by two numbers *(position, id)*: the position where it begins, and the identifier of the founding haplotype from which it is derived. This cartoon depicts a chromosome with 3 haplotype chunks as the result of recombination (double crossover) between two founder chromosomes.

`forqs` begins with a set of founding haplotypes representing the individuals in the initial generation. By assigning a unique identifier to each founding haplotype, individual haplotype chunks are tracked as they recombine over subsequent generations. For the purposes of simulation, any existing neutral variation on the haplotype chunks can be ignored. When simulating selection on standing variation, only those loci with fitness effects need to be tracked.

Internally, `forqs` represents an individual chromosome as a list of haplotype chunks (Figure 1). Individuals are diploid, and carry a user-specified number of chromosome pairs. To represent genetic variation at particular loci, `forqs` queries functions called `VariantIndicators` to obtain the variant values carried by the founding haplotypes at those loci.

`forqs` uses configurable *modules* (Section 4) that are plugged into the main simulator to change the behavior of the simulation (Figure 2). `forqs` performs the following actions during a single cycle of the simulation:

(1-3) Generation of new populations: The `PopulationConfigGenerator` module (1) provides the simulator with a *population configuration* that specifies how to create individuals in the next generation from parents in the previous generation, based on user-specified population size and migration rate trajectories. Recombination and mutation are handled by a user-specified `RecombinationPositionGenerator` module (2) and/or `MutationGenerator` module (3), respectively.

(4) Genotyping: individuals are "genotyped" at a set of loci, using the `VariantIndicator` module to obtain variant (SNP) values for each individual. The list of loci to genotype is determined from the quantitative traits and reporters that are specified by the user.

(5) Quantitative trait evaluation: for each quantitative trait, a trait value is calculated for each individual, based on the individual's genotype at loci

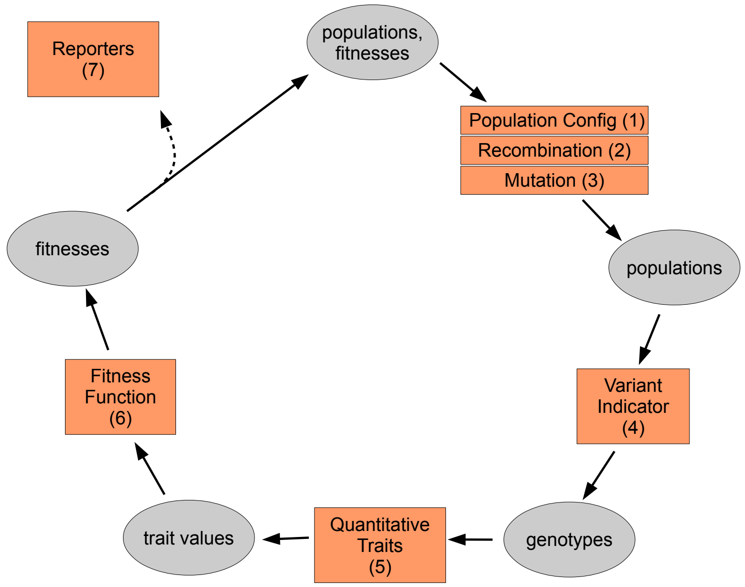

Figure 2: **`forqs` modular design**. The orange boxes represent places where the user can plug in configurable modules.

affecting the trait. Each quantitative trait is specified by a `QuantitativeTrait` module.

(6) Fitness evaluation: the `FitnessFunction` module calculates each individual's fitness based on the individual's quantitative trait values.

(7) Reporting: each `Reporter` module updates its output files using current information from the populations (which includes genotypes, trait values, and fitnesses).

Each configurable module in Figure 2 is actually an interface with multiple implementations. The user specifies which module implementations to instantiate in a `forqs` configuration file. More details on the primary module interfaces and their interaction with the simulator can be found in Section 4.4. The `forqs` Module Reference contains detailed information on the primary module implementations available to the user.

In addition to the primary modules shown in the diagram, there are several building block modules (Section 4.5) that provide basic functionality to the primary modules. For example, `Trajectory` modules provide a unified method for specifying values that change over time, such as population sizes or migration rates. Similarly, `Distribution` modules can be used to specify how to draw particular random values, for example, QTL positions or allele frequencies.

3 Tutorial Introduction

In this section we introduce `forqs` configuration files, starting with a minimal example and gradually adding modules to add complexity to the simulation. All example configuration files can be found in the `examples` directory of the `forqs` package.

3.0 A minimal example

We start with a minimal example configuration file:

```
#
# tutorial_0_minimal.txt
#

PopulationConfigGenerator_ConstantSize pcg
    population_size = 10
    generation_count = 3

SimulatorConfig
    output_directory = output_tutorial_0_minimal
    population_config_generator = pcg
```

There are two modules specified in this configuration file. The first module is `PopulationConfigGenerator_ConstantSize`, which we have assigned the object id `pcg`. The object id, which can be any text string, allows other objects to reference this object. We have set two parameters for this module, which specify the population size (`population_size = 10`) and the number of generations to simulate (`generation_count = 3`).

The second module is `SimulatorConfig`, the top-level module. This module must be specified last in the configuration file, because it has references to the primary modules (the general rule is: objects must be specified before they can be referenced by other objects). In this case, we are telling `SimulatorConfig` to use object `pcg` as our `PopulationConfigGenerator`, which is the only primary module that is required to be specified. The other primary modules have trivial defaults, and we leave them unspecified. In addition to the primary module references, `SimulatorConfig` also contains global simulation parameters. In this case, we have specified the output directory to be `output_tutorial_0_minimal`.

The simulator queries the `PopulationConfigGenerator` each generation to obtain a population configuration, which contains the information necessary to create the next generation from the previous one. In this case, the population configuration is the same each generation — it tells the simulator to create a population of 10 individuals with parents drawn from the same population in the previous generation.

When you run `forqs` on this example (`tutorial_0_minimal.txt`), you will see screen output showing that the simulation ran for 3 generations. You will also see the new output directory `output_tutorial_0_minimal`. If you look in this directory, you'll see a single file: `forqs.simconfig.txt`. This file contains

the configuration used for the simulation, including unspecified default parameters. However, there are no other output files in the directory, so we don't actually know what happened during the simulation. To get more information, we need to specify Reporters to report output — we show how to do this in the next section.

3.1 Recombination and reporting output

In this example, we add modules to include recombination and report output:

```
#
# tutorial_1_recombination_reporter.txt
#

PopulationConfigGenerator_ConstantSize pcg
    generation_count = 3
    population_count = 1
    population_size = 10
    chromosome_lengths = 1e6

RecombinationPositionGenerator_Uniform rpg
    rate = 1

Reporter_Population reporter_population
    # update_step = 1

SimulatorConfig
    output_directory = output_tutorial_1_recombination_reporter
    population_config_generator = pcg
    recombination_position_generator = rpg
    reporter = reporter_population
```

When creating offspring chromosomes from parental chromosomes, the simulator uses the `RecombinationPositionGenerator` module to generate lists of recombination positions. In this case, we are using `RecombinationPositionGenerator_Uniform`, which chooses the positions uniformly at random along the chromosome. Note the use of the compound parameter `chromosome_length_rate` that specifies both the length of the chromosome and the recombination rate.

`Reporter` modules are used by `forqs` to output information about the simulation. The user can specify an arbitrary number of `Reporters`, depending on what output is desired. `Reporter_Population` outputs `forqs` population files, where each individual is represented as a list of chromosomes (one chromosome per line) and each chromosome is represented as a list of haplotype chunks. By default, population files will be produced only for the final generation — setting `update_step = n` will tell the `Reporter_Population` to output population files every `n` generations. You can see this by uncommenting (removing the '#' character) the `update_step` line in the configuration file and re-running the simulation.

The population files contain the raw information about the simulated individuals, and are not meant to be analyzed directly. Instead, they can be used to propagate existing neutral variation from the founding haplotypes of the initial generation to the mosaic haplotypes of the final generation (see Section 5).

However, we will use the population files to illustrate `forqs`' internal representation of chromosomes. The first chromosome in the population file from the final generation is:

```
+ { (0,12) (704958,7) }
```

This means that the first chromosome of the first individual is made up of two haplotype chunks: the first chunk (0,12) starts at position 0 and comes from founder individual 12; the second chunk (704958,7) starts at position 704958 and comes from founder individual 7. Now comment out the following line in `SimulatorConfig`:

```
    recombination_position_generator = rpg
```

which 'un-plugs' the `RecombinationPositionGenerator` from the simulator. Now re-run the simulation, specifying a different output directory on the command line:

```
    forqs tutorial_1_recombination_reporter.txt output_directory=out2
```

When you look at the resulting final population file, you will see that all chromosomes consist of a single chunk, as you would expect with no recombination.

3.2 Wright-Fisher simulation

Our next example is a simple Wright-Fisher simulation where we track the allele frequency at a single locus under neutral drift:

```
#
# tutorial_2_wright_fisher.txt
#

PopulationConfigGenerator_ConstantSize pcg
    population_size = 100
    generation_count = 200
    # population_count = 10

Locus my_locus
    chromosome = 1
    position = 100000

VariantIndicator_SingleLocusHardyWeinberg vi
    locus = my_locus
    allele_frequency = .5

Reporter_AlleleFrequencies reporter_allele_freqs
    locus = my_locus
```

```
SimulatorConfig
    output_directory = output_tutorial_2_wright_fisher
    population_config_generator = pcg
    variant_indicator = vi
    reporter = reporter_allele_freqs
```

In this example, the population size is 100, the initial allele frequency is .5, and the simulation runs for 200 generations.

The `Locus` module defines a single site — in this case it is position 100000 on chromosome 1. The `Locus` has id `my_locus`, and this id is used by two other modules to refer to this locus.

As we saw in the previous example, `forqs` represents chromosomes as lists of haplotype chunks, with no information about nucleotides or variant (SNP) values at any position. (To be concrete, we think of a variant value as being 0 or 1, but `forqs` variant values can be anything in the range 0–255, so we are not restricted to bi-allelic SNPs). However, if we give `forqs` the variant values for the founding haplotypes, it can calculate variant values for any mosaic chromosome. This information is represented by a `VariantIndicator`, which is a function that tells `forqs` which founding chromosomes contain which variants.

In our case, we specify a `VariantIndicator_SingleLocusHardyWeinberg`, which assigns variants to individuals in Hardy-Weinberg proportions. Because we have specified an initial allele frequency of .5, this means that of the founding individuals, 25% will be homozygote 0, 25% will be homozygote 1, and 50% will be heterozygotes.

We also want to track the allele frequency at this locus, so we specify a `Reporter_AlleleFrequencies`, which reports the allele frequency at each generation. You will find the output in the file `allele_frequencies_chr1_pos100000.txt`.

You may have noticed the commented line with the parameter `population_count = 10`. By uncommenting this line, you will tell `forqs` to simulate 10 populations. `PopulationConfigGenerator_ConstantSize` does not allow migration between populations, so this effectively gives 10 independent Wright-Fisher simulations. The resulting allele frequency trajectories can all be found in the same output file, with one column per population. As expected, you will see that the alternate allele (1) has been fixed or lost in some populations, and is still polymorphic in others.

3.3 Selection

In this example, we add selection to our Wright-Fisher simulation.

```
#
# tutorial_3_selection.txt
#

PopulationConfigGenerator_ConstantSize pcg
```

```
    population_size = 100
    generation_count = 100
    population_count = 10

Locus my_locus
    chromosome = 1
    position = 100000

VariantIndicator_SingleLocusHardyWeinberg vi
    locus = my_locus
    allele_frequency = .5

QuantitativeTrait_SingleLocusFitness qt
    locus = my_locus
    w0 = 1
    w1 = 1.1
    w2 = 1.2

FitnessFunction_Identity ff
    quantitative_trait = qt

Reporter_AlleleFrequencies reporter_allele_freqs
    locus = my_locus

Reporter_MeanFitnesses reporter_mean_fitnesses

Reporter_DeterministicTrajectories reporter_deterministic_trajectories
    initial_allele_frequency = .5
    w0 = 1
    w1 = 1.1
    w2 = 1.2

SimulatorConfig
    output_directory = output_tutorial_3_selection
    population_config_generator = pcg
    variant_indicator = vi
    quantitative_trait = qt
    fitness_function = ff
    reporter = reporter_allele_freqs
    reporter = reporter_mean_fitnesses
    reporter = reporter_deterministic_trajectories
```

For this simulation, we add a quantitative trait that is determined by the genotype at a single locus. In fact, in our case, the quantitative trait *is* fitness (`QuantitativeTrait_SingleLocusFitness`), and we use the identity function for our fitness function (`FitnessFunction_Identity`). The parameters `w0, w1, w2` are used to specify the fitness for individuals with genotype 0, 1, and 2, respectively. In general, a quantitative trait can depend on multiple loci on multiple chromosomes, and fitness can depend on multiple quantitative traits.

We also add some useful `Reporters` that output the mean fitnesses of the populations as well as the deterministic trajectories expected in the limit of infinite population size. These output files can be easily read into a plotting application (e.g. `R`) to compare the random trajectories with the deterministic trajectories (Figure 3).

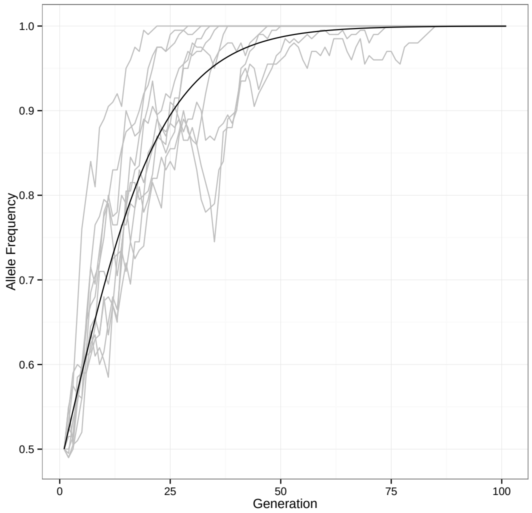

Figure 3: **Allele frequency trajectories from selection simulation**.

3.4 Trajectories

This section shows how to use `Trajectory` modules to specify population sizes that change over time. In general, `Trajectory` modules can be used to specify any numeric value that varies by population and generation – other examples include mutation rates and migration rates.

In the first example (`tutorial_4a_trajectories.txt`), there are two populations following the same population size trajectory: constant size 100 until generation 2, followed by a linear increase in population size until it reaches 400 at generation 5, after which the population size stays at 400. This is accomplished by specifying each piece (`Trajectory_Constant`, `Trajectory_Linear`, `Trajectory_Constant`) and composing the pieces together with `Trajectory_GenerationComposite`.

```
#
# tutorial_4a_trajectories.txt
#
```

```
Trajectory_Constant popsize_1
    value = 100

Trajectory_Linear popsize_2
    begin:value = 2 100
    end:value = 5 400

Trajectory_Constant popsize_3
    value = 400

Trajectory_GenerationComposite popsize
    generation:trajectory = 0 popsize_1
    generation:trajectory = 2 popsize_2
    generation:trajectory = 5 popsize_3

PopulationConfigGenerator_LinearSteppingStone pcg
    generation_count = 8
    population_count = 2
    population_size = popsize

SimulatorConfig
    output_directory = output_tutorial_4a_trajectories
    write_popconfig = 1        # set this flag to write out the popconfig file
    population_config_generator = pcg
```

Note that `PopulationConfigGenerator_LinearSteppingStone` has a `population_size` parameter whose value must be the id of a `Trajectory` module (rather than a numeric value). Also, unless otherwise specified, each `Trajectory` gives the same numeric value for each population in a given generation. You can see the raw population configuration generated by including the parameter `write_popconfig = 1`, which tells `forqs` to write out the population configs in a file `forqs.popconfig.txt`. After running this example, you can examine `forqs.popconfig.txt` to see the population sizes.

In the next example (`tutorial_4b_trajectories.txt`), population 1 follows the same population size trajectory as in the first example, while population 2 experiences exponential growth, doubling each generation (with initial population size 1000). In order to specify this scenario, each population size trajectory is specified separately (`popsize_pop1` and `popsize_pop2` in this example), and the two are composed with `Trajectory_PopulationComposite`. When `Trajectory_PopulationComposite` is queried for a value for a population, it returns the value obtained from the appropriate sub-trajectory.

```
#
# tutorial_4b_trajectories.txt
#

# population 1
```

```
Trajectory_Constant popsize_pop1_1
    value = 100

Trajectory_Linear popsize_pop1_2
    begin:value = 2 100
    end:value = 5 400

Trajectory_Constant popsize_pop1_3
    value = 400

Trajectory_GenerationComposite popsize_pop1
    generation:trajectory = 0 popsize_pop1_1
    generation:trajectory = 2 popsize_pop1_2
    generation:trajectory = 5 popsize_pop1_3

# population 2

Trajectory_Exponential popsize_pop2
    generation_begin = 0
    value_begin = 1000
    rate = 0.693147     # log(2); population size doubles each generation

# composite trajectory

Trajectory_PopulationComposite popsize
    trajectories = popsize_pop1 popsize_pop2

PopulationConfigGenerator_LinearSteppingStone pcg
    generation_count = 8
    population_count = 2
    population_size = popsize

SimulatorConfig
    output_directory = output_tutorial_4b_trajectories
    write_popconfig = 1        # set this flag to write out the popconfig file
    population_config_generator = pcg
```

3.5 Quantitative traits

In this section, we present an example of specifying a quantitative trait by specifying quantitative trait loci (QTLs) and their effect sizes.

```
#
# tutorial_5_qtl.txt
#

PopulationConfigGenerator_ConstantSize pcg
    generation_count = 10
    population_count = 1
```

```
    population_size = 100
    chromosome_pair_count = 3
    chromosome_lengths = 1e6 1e6 1e6

Locus locus1
    chromosome = 1
    position = 1000

Locus locus2
    chromosome = 2
    position = 2000

Locus locus3
    chromosome = 3
    position = 3000

LocusList loci
    loci = locus1 locus2 locus3

VariantIndicator_Random vi
    locus_list:population:frequencies = loci * .5 .5 .5

QuantitativeTrait_IndependentLoci qt
    qtl = locus1 0 .1 .2
    qtl = locus2 0 .2 .4
    qtl = locus3 0 .1 .2
    environmental_variance = .05

FitnessFunction_TruncationSelection ff
    quantitative_trait = qt
    proportion_selected = .5

Reporter_AlleleFrequencies reporter_allele_frequencies
    quantitative_trait = qt

Reporter_TraitValues reporter_trait_values
    quantitative_trait = qt
    write_full = 1

SimulatorConfig
    output_directory = output_tutorial_5_qtl
    population_config_generator = pcg
    variant_indicator = vi
    quantitative_trait = qt
    fitness_function = ff
    reporter = reporter_allele_frequencies
    reporter = reporter_trait_values
    write_vi = 1
```

In this example, we specify a single population (size 100) where individuals have 3 chromosomes. We specify 3 loci, each on a different chromosome.

VariantIndicator_Random is used to assign variants to the initial haplotypes randomly, according to the specified allele frequencies – in this case, the variant allele frequency is .5 for each locus. The '*' indicates that all populations have the same allele frequencies – because there is only one population, we could have used '1' in place of '*'. The parameter write_vi = 1 in SimulatorConfig tells forqs to write the file forqs.vi.txt containing lists of the initial haplotypes carrying the variant allele at each locus.

QuantitativeTrait_IndependentLoci specifies a quantitative trait where the effects of variants at different loci are independent of each other, i.e. there are no epistatic effects. Each QTL is listed with its per-genotype effects – in this case, individuals with genotype 0, 1, or 2 at locus1 will have 0, .1, or .2 added to their trait value, respectively. Alternatively, QTL effect sizes and dominance values can be chosen randomly according to specified distributions. For an example of this, see example_qtl_random.txt which also demonstrates the use of LocusList_Random to pick the loci randomly as well.

In this example, FitnessFunction_TruncationSelection assigns fitness values of 1 to individuals in the upper half of the distribution, and 0 to individuals in the lower half (proportion_selected = .5). Reporter_AlleleFrequencies uses the parameter quantitative_trait = qt, which tells it to report allele frequencies for all QTLs associated with the trait. Reporter_TraitValues reports mean trait values for the population each generation; the parameter write_full = 1 tells it to also report individual trait values for each generation.

3.6 Reporting haplotype frequencies

This example shows the use of Reporter_HaplotypeFrequencies to report local haplotype frequencies in the simulated populations.

In this example, there is a single locus that confers an additive fitness advantage to the individuals carrying the selected variant. The VariantIndicator specifies which haplotype ids actually carry the variant: in this case, VariantIndicator_IDRange assigns the value 1 to the first 1000 haplotype ids, i.e. the first 500 individuals are homozygous for the selected variant.

Reporter_HaplotypeFrequencies uses a HaplotypeGrouping module that specifies how haplotype ids should be grouped. The grouping can be as fine-grained as a single haplotype id per group, but for simplicity we have specified just two groups: those individuals who carry the selected variant, and those who don't. Reporter_HaplotypeFrequencies then reports local proportions of the two groups in the population.

We have specified a relatively high recombination rate (3 crossovers per meiosis on average) and strong selection (.1 selection coefficient) in order to illustrate the change in local haplotype frequencies in just a few generations. After running the example, the output file haplotype_frequencies_chr1_final_pop1.txt shows that haplotypes carrying the selected variant have risen to high fre-

quency near the selected locus, but not as much in regions farther away, due to recombination. The parameter `update_step = k` will result in reporting of local haplotype frequencies every `k` generations (default is to report only the final generation).

```
#
# tutorial_6_haplotype_frequencies.txt
#

PopulationConfigGenerator_ConstantSize pcg
    generation_count = 20
    population_count = 1
    chromosome_pair_count = 1
    chromosome_lengths = 1e6
    population_size = 1000

RecombinationPositionGenerator_Uniform rpg
    rate = 3

Locus selected_locus
    chromosome = 1
    position = 500000

VariantIndicator_IDRange vi
    locus:start:count:step:value = selected_locus 0 1000 1 1

QuantitativeTrait_IndependentLoci qt
    qtl = selected_locus 1 1.1 1.2

FitnessFunction_Identity ff
    quantitative_trait = qt

Reporter_AlleleFrequencies reporter_allele_frequencies
    quantitative_trait = qt

HaplotypeGrouping_Uniform hg
    ids_per_group = 1000        # 2 groups of 500 individuals

Reporter_HaplotypeFrequencies reporter_haplotype_frequencies
    haplotype_grouping = hg
    chromosome_step = 1e5
    #update_step = 1

SimulatorConfig
    output_directory = output_tutorial_6_haplotype_frequencies
    population_config_generator = pcg
    recombination_position_generator = rpg
    variant_indicator = vi
    quantitative_trait = qt
    fitness_function = ff
```

```
reporter = reporter_allele_frequencies
reporter = reporter_haplotype_frequencies
```

4 Modules

In this section, we give an overview of the forqs module interfaces. For information about specific forqs module implementations, including parameter names, usage, and links to examples, please refer to the forqs Module Reference (forqs_module_reference.html).

4.1 Module types and interfaces

forqs has a single *top-level module* called SimulatorConfig. There are seven *primary modules*, corresponding to the orange boxes Figure 2, which represent the main places where forqs can be customized. There are also several *building block modules*, which are used by the primary modules.

Multiple modules may share a common interface, as indicated by their names. For example, Reporter_Timer and Reporter_AlleleFrequencies both implement the Reporter interface. Modules with the same interface can be used interchangeably by the simulator.

In addition, specific modules can be instantiated multiple times (with different object ids and parameters) – for example, Reporter_AlleleFrequencies can be used to report allele frequencies at several different loci.

By mixing and matching different modules, the user has a great deal of flexibility in creating customized simulation scenarios. In addition, forqs can easily be extended with new functionality by adding new modules that implement existing module interfaces.

4.2 Module specification and instantiation

Each forqs module specified in the configuration file corresponds to an object that is instantiated by the simulator before the simulation starts. The first line of a module specification contains the module name and object id. Each subsequent line specifies a parameter of the module, formatted as a name-value pair: *name = value*. The value is read as a string, but may be interpreted as an integer, floating point, string, or list, depending on the parameter. Each module determines how its parameter values are interpreted, and interpretation errors are reported to the user. In some modules, multiple parameter values may be specified with the same name, in which case each value specified is appended to a list. A blank line ends the specification for that module.

As an example, here is the specification of a Locus module, which represents a single nucleotide position:

```
Locus my_locus
    chromosome = 1
    position = 500000
```

The first line specifies a Locus object with id my_locus. The two parameters specify which chromosome (1) and the position on the chromosome (500000).

After parsing this module specification, the simulator instantiates a `Locus` object, and puts the object in a registry under the name `my_locus`. Subsequently instantiated objects may need a reference to this object, which can be obtained by looking up the object by name in the registry. Because modules are instantiated in the order they are specified in the configuration file, it is important to specify an object before it is referenced by other objects (otherwise `forqs` will produce an error message that it couldn't find the referenced object in the registry).

Object references are specified as string-valued parameters, where the value contains the object id of the referenced object. For example, the following is the specification of a `Reporter` module that holds a reference to our example `Locus` object `my_locus`:

```
Reporter_AlleleFrequencies reporter_allele_frequencies
    locus = my_locus
```

One final note on a parameter naming convention used in `forqs` – for parameters where the value is a list of sub-parameters, the parameter name should be a concatenation of the sub-parameters. This convention allows easier reading and editing of configuration files without the need to consult documentation. For example, `Trajectory_GenerationComposite` represents a list of `Trajectory` modules, to be followed piecewise at specified generations. This module is parametrized by a list of pairs (generation, trajectory), using the parameter name `generation_trajectory`:

```
Trajectory_GenerationComposite id_migration_rate
    generation_trajectory = 0 id_migration_rate_0
    generation_trajectory = 5 id_migration_rate_1
```

In this case, the module configuration can be interpreted as follows: at generation 0 use trajectory `id_migration_rate_0`, and then at generation 5 start using trajectory `id_migration_rate_1`.

4.3 Top-level module: SimulatorConfig

`SimulatorConfig` is the top-level module containing:

- global simulation parameters
- references to the primary modules

Because `SimulatorConfig` has references to the primary modules, it must be specified last in the configuration file.

4.3.1 Global simulation parameters

The main global simulation parameters are:

- `output_directory`: `forqs` will create this directory and place all output files here

- `seed`: seed for the random number generator

Command line parameters can also be specified on the command line as *name=value* with no whitespace, as shown in this example:

```
forqs config.txt output_directory=mydir seed=12345
```

If a parameter is specified on both the command line and in the configuration file, the command line takes precedence.

If no random seed is specified, `forqs` will first look in the current working directory for the file `forqs.seed` containing a previously generated seed. If no seed is found, `forqs` will generate a new seed from the system time. At the end of each run, `forqs` generates a random seed and writes it to `forqs.seed`. To summarize, `forqs` looks for the seed in the following places, in order of preference:

1. command line
2. configuration file
3. `forqs.seed`
4. system time

4.3.2 References to primary modules

Of the primary modules, only `PopulationConfigGenerator` is required to be specified, because it contains necessary information about population sizes and the number of generations. The rest of the modules default to trivial implementations. The primary modules specified in `SimulatorConfig` are:

1. `PopulationConfigGenerator`
    - required – no default
2. `RecombinationPositionGenerator`
    - default: `RecombinationPositionGenerator_Trivial` (no recombination – whole chromosomes are transmitted, chromosome pairs segregate independently)
3. `MutationGenerator`
    - default: none (no mutation)
4. `VariantIndicator`
    - default: `VariantIndicator_Trivial` (always returns 0)
5. `QuantitativeTrait`
    - default: none (no quantitative traits)

- multiple QuantitativeTraits may be defined

6. FitnessFunction
   - default: FitnessFunction_Trivial (always returns 1 – all individuals have equal fitness)

7. Reporter
   - default: none (no reporters)
   - multiple Reporters may be defined

4.4 Primary modules

4.4.1 PopulationConfigGenerator

For the reproduction/transmission step, in addition to information about the current populations, the simulator needs to know the *population configuration* for the next generation, which includes:

- number and sizes of the populations in the next generation
- mating distribution, which describes how parents are chosen from populations in the current generation to create offspring in the next generation

The PopulationConfigGenerator module provides an interface for the simulator to obtain a population configuration for each generation. To create each new individual in the next generation, the simulator chooses parent populations according to the mating distribution, and then individual parents with probability proportional to their fitnesses.

For the initial generation, individuals are assigned haplotype ids sequentially starting at 0, with two ids assigned per individual (one for the maternal chromosomes, and one for the paternal chromosomes). Id offsets can be used to make it easier to distinguish between populations – for example, individuals from population 1 may be assigned ids starting at 0, and individuals from population 2 may be assigned ids starting at 1000000.

PopulationConfigGenerator_File allows the user to specify a population configuration for each generation, giving the user precise control over the demographic histories of the simulated populations.

Alternatively, forqs provides higher level PopulationConfigGenerators that allow the user to specify time-dependent population size and migration rate trajectories (e.g. PopulationConfigGenerator_LinearSteppingStone). When a migration rate is specified from a source population to a destination population, it is interpreted to be the probability that a new child in the destination population has parents in the source population. Equivalently, it is the expected proportion of individuals in the next generation of the destination population whose parents were in the source population.

For debugging purposes, forqs optionally outputs the file forqs.popconfig.txt with the population configuration that was used for each generation during

the simulation. This option can be selected by setting the `write_popconfig` parameter in `SimulatorConfig`:

```
write_popconfig = 1
```

4.4.2 RecombinationPositionGenerator

The `RecombinationPositionGenerator` module provides an interface for the simulator to obtain a list of recombination positions. After querying the `RecombinationPositionGenerator`, the simulator uses the list of recombination positions to create an offspring chromosome from a pair of parental chromosomes.

There are multiple implementations of the `RecombinationPositionGenerator` module interface, each of which uses a different method to generate the recombination position list.

`RecombinationPositionGenerator_SingleCrossover` assumes there is a single crossover event at a position chosen uniformly at random over that chromosome. This results in the offspring receiving one of the two parental or one of the two recombined chromosomes with equal 25% probabilities.

`RecombinationPositionGenerator_Uniform` generates recombination positions according to a uniform Poisson process along the chromosome, with rate specified by the user.

`RecombinationPositionGenerator_RecombinationMap` generates recombination positions according to previously estimated recombination maps, in the format used by the HapMap project.

4.4.3 MutationGenerator

The user may specify a `MutationGenerator` module to generate random mutations during the simulation. `MutationGenerator` provides a single interface through which the simulator obtains a list of new mutations for a particular generation. However, `MutationGenerator` implementations may differ in how they generate this list of mutations. For example, `MutationGenerator_SingleLocus` generates mutations only at a single site, while `MutationGenerator_Regions` generates mutations in multiple regions with different (possibly time-dependent) mutation rates. In any case, the user will most likely want to also specify `Reporter_Regions` to report the final mutated sequences at the end of the simulation.

`forqs` current mutation implementation creates a new haplotype id for each new mutation, storing it in a haplotype ancestry tree (so that information about the ancestral haplotype can be preserved). This results in memory usage that increases with each generation, which may cause performance degradation in simulations involving a high total mutation rate for a large number of generations. (Here *total mutation rate* means θL, where $\theta = 4N\mu$ is the population-scaled per-site mutation rate and L is the total length of the genomic region where new mutations are being generated).

4.4.4 VariantIndicator

Internally, `forqs` represents each individual chromosome as a list of haplotype chunk ids, with no information about particular variants carried on that chromosome. In order to obtain an individual's genotype at a locus, the simulator must use a `VariantIndicator`, which is essentially a function that maps:

$$(locus, haplotype\ id) \mapsto variant\ value$$

Typically these variant values will be 0/1 for SNPs; however, any value in the range 0-255 can be used.

In general, a `VariantIndicator` needs to specify variant values only at selected loci, because neutral loci have no effect on the simulation. Ancestral neutral variation that is present on the haplotypes of the founders (individuals in the initial generation) can be propagated to individuals at the end of the simulation using the `forqs_map_ms` tool included in the `forqs` package.

Several `VariantIndicator` implementations are available, allowing the user a variety of options for specifying the variants carried by the founders – these can be found in the `forqs` Module Reference.

4.4.5 QuantitativeTrait

A `QuantitativeTrait` module represents a single quantitative trait. The module encapsulates the information needed to calculate the trait value for each individual based on the individual's genotypes at a list of user-specified loci. For example, `QuantitativeTrait_IndependentLoci` allows the user to specify loci and effect sizes, and the trait value for each individual is calculated by combining that individual's locus-specific effects additively (with user-specified environmental variance). Full documentation on the available `QuantitativeTraits` can be found in the `forqs` Module Reference.

4.4.6 FitnessFunction

A `FitnessFunction` module represents the function used to calculate an individual's fitness based on the individual's trait values. In simple cases, the quantitative trait may actually be fitness, in which case the user may simply use `FitnessFunction_Identity`. In more complicated cases, fitness may depend non-trivially on one or more quantitative traits. Full documentation on the available `FitnessFunctions` can be found in the `forqs` Module Reference.

4.4.7 Reporter

`Reporter` modules are used to report information during the simulation. For example, `Reporter_AlleleFrequencies` reports the allele frequency of a user-specified locus at each generation. The full list of available `Reporters` can be found in the `forqs` Module Reference.

4.5 Building block modules

4.5.1 Locus and LocusList

The `Locus` module represents a single site on a chromosome, specified by the chromosome pair index and position on the chromosome:

```
Locus my_locus
    chromosome_pair_index = 0
    position = 500000
```

Note that the chromosome pairs use 0-based indexing, so `chromosome_pair_index = 0` refers to the first chromosome pair.

`LocusList` modules are used to define a list of loci. The list can be specified in two ways – in the first, loci are specified by chromosome number and position:

```
LocusList locus_list
    chromosome:position = 1 1000123
    chromosome:position = 2 2000234
    [...]
```

Alternatively, the loci can be references to previously specified `Locus` objects:

```
LocusList locus_list_2
    loci = my_locus_1 my_locus_2 [...]
```

Also, a random list of loci can be generated with `LocusList_Random`:

```
LocusList_Random locus_list_random
    locus_count = 10
```

4.5.2 Trajectory

In simple situations, constant parameter values can be used to specify an aspect of the simulation. For example, it is common in population genetics to simulate models where population sizes are constant. In more complicated scenarios, parameter values (e.g. population sizes, migration rates, mutation rates, optimal quantitative trait values) may vary in space (population) or in time (generation).

`forqs` uses `Trajectory` modules to describe such varying quantities. In essence, a `Trajectory` represents a function:

$$(\textit{population index}, \textit{generation index}) \mapsto \textit{value}$$

Trajectories can be built by the user by starting with simple building blocks (e.g. constant, linear, polynomial, exponential functions) and then composed either by population or by generation. Once the `Trajectory` has been defined, it can be referenced by name to be used as the parameter of another module. Note that if a module expects a particular parameter to be a `Trajectory`, it is an error to pass a constant integer or floating point number as the parameter value. In this case, if the user really wants a constant parameter value, `Trajectory_Constant` should be specified.

4.5.3 Distribution

`Distribution` modules represent probability distributions. These modules are used to model, for example, effect sizes for QTLs or environmental variance for a quantitative trait.

`Distribution_Constant` can be used in situations where a module requires a reference to a `Distribution` but a constant value is desired.

5 Simulating background variation

Forward-in-time simulators often use a burn-in period to allow neutral variation to reach mutation-drift equilibrium. An alternative strategy, adopted by `forqs`, is to use an existing program (such as Dick Hudson's `ms`) to generate neutral variation for the founders in the initial population. Chromosomes in the final populations are mosaics of the founder chromosomes, so neutral variation can be propagated to the mosaic chromosomes to generate sequence (or SNP) data. The tool `forqs_map_ms` is included in `forqs` packages for this purpose. In this section we discuss the propagation procedure using two examples.

5.1 Example 1: No new mutations

The first example is a simple case where there are no new mutations introduced during the `forqs` simulation. This case includes many scenarios of interest where the variation in the population has been generated over a small number of generations, primarily by recombination and recent admixture between historically isolated (or inbred) populations.

In this case, the propagation of neutral variation is straight-forward: sequence variants on founder haplotypes are mapped onto the mosaic chromosomes of the final population. However, there are many details about the mapping that must be specified by the user in a mapping configuration file. `forqs` produces final populations of individuals that may carry multiple pairs of chromosomes, with absolute positions specified by integers. `ms` outputs single chromosomes, with relative positions specified by floating point numbers. The mapping configuration thus needs to specify an integer range of positions on a particular chromosome that correspond to the `ms` relative positions in the range [0,1]. In addition, the user must specify how `forqs` haplotype ids correspond to the `ms`-format variant sequences.

In the `examples` directory, the following 4 files can be found:

- `population_example.txt` (`forqs` population data)
- `ms_test_data_1.txt, ms_test_data_2.txt` (`ms`-format files)
- `ms_map_config.txt` (mapping configuration file)

Perform the mapping using the following command:

```
forqs_map_ms population_example.txt ms_map_config.txt > output.ms
```

The resulting sequences in `output.ms` are mosaics of the sequences in the input `ms`-format files. For example, the first chromosome of the first individual is a mosaic of haplotypes 0, 1, 2, and 3, corresponding to the sequences 'aaaaaaaaa', 'bbbbbbbbb', 'ccccccccc', and 'ddddddddd', respectively. The output sequence for this chromosome is 'aabccccdd'. Note that alphabetic letters are used in these example sequences for illustration only – real `ms` output consists of 0's and 1's.

5.2 Example 2: Including new mutations

In scenarios where it is important to include new mutations in addition to ancestral neutral variation, the mapping procedure has one additional complication. When simulating forward in time, the neutral variants carried by individuals have no effect on the dynamics of the simulation. `forqs` takes advantage of this by ignoring (i.e. not storing in memory) any ancestral neutral variation on the founding haplotypes. Each new mutation generated in `forqs` results in a new haplotype id. Because of this, it is necessary to use an id ancestry map to translate the new ids back to the ancestral ids before performing the mapping. After the neutral variation has been mapped, the new mutations can then be merged with the ancestral variants. This procedure is detailed in the following shell script (and data files referenced in the script), which runs a complete example of a `forqs` simulation followed by mapping `ms` sequences (with new mutations merged):

```
examples/ms_map_example_new_mutations.sh
```

6 Validation

`forqs` has an extensive set of unit tests that verify the correctness of individual code modules. In addition, in order to validate the larger-scale behavior of the simulations, we have compared `forqs` simulation results to theoretical predictions from population genetics and quantitative genetics.

6.1 Single locus selection

We first considered simple scenarios where an individual's fitness is determined by that individual's genotype at a single locus, under a wide range of fitness effect sizes and dominance values. We compared the simulated allele frequency trajectories to the deterministic trajectories predicted in the limit of infinite population size.

In all cases, the simulated trajectories closely followed the deterministic trajectories, with better agreement in simulations with larger population sizes, as expected. Figure 4 shows the case of additive positive selection, for population sizes of 100 and 1000.

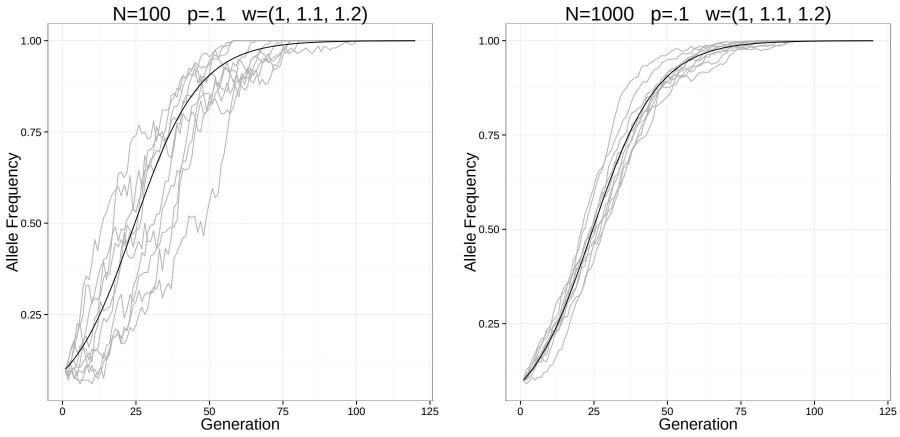

Figure 4: **Selection on a single locus**. The dark curves show the deterministic trajectories, and the lighter curves show simulated trajectories. Populations of size 100 and 1000 were simulated with additive fitness effect .1 at a single locus, with the selected variant having initial allele frequency .1.

6.2 Decay of linkage disequilibrium

In the limit of infinite population size, linkage disequilibrium (measured by D, the correlation between two variants at different sites) decays geometrically at rate $1-r$, where r is the recombination rate (see Figure 5 for an example run).

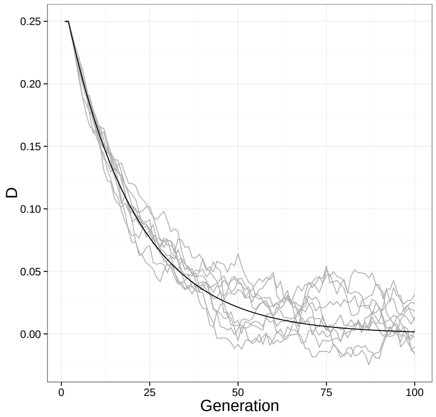

Figure 5: **Decay of linkage disequilibrium**. The dark curve shows the deterministic trajectory, and the lighter curves show simulated trajectories. In this example, $D = .25$ initially and $r = .05$.

6.3 Mutation-drift equilibrium

To validate our mutation implementation, we approximated the infinite-sites model by simulating mutations generated randomly in large regions (1mb). We set the per-site mutation rate to make the population-scaled mutation rate $\theta = 10$, and simulated for enough generations to reach mutation-drift equilibrium.

We then compared the resulting site-frequency spectra $\{\xi_i\}$ to theoretical predictions, where ξ_1 is the number of singletons, ξ_2 is the number of doubletons, etc. in the sample. Coalescent theory predicts that $\xi_i = \theta/i$ in expectation (Fu 1995), under the assumption that the sample size is small compared to the population size. When the sample size is close to the population size, the Wright-Fisher model is expected to have approximately 12% more singletons and 2% less doubletons compared to the coalescent expected values (Wakeley and Takahashi 2003).

Site frequency spectra generated from the full population agree with the Wright-Fisher large sample expected values, and site frequency spectra from smaller samples agree with the coalescent expected values, as expected (Figure 6).

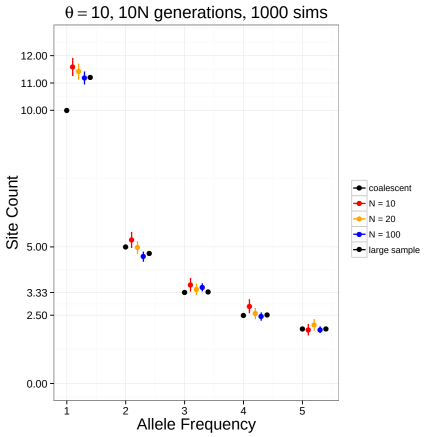

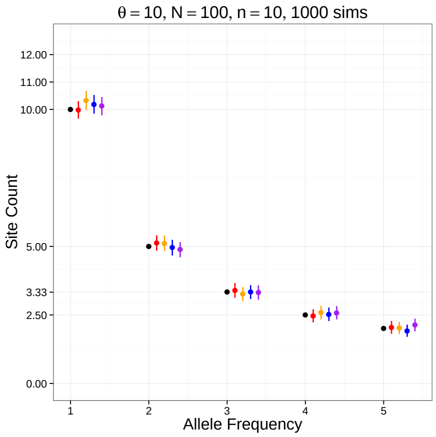

Figure 6: **Mutation-drift equilibrium**. (*left*) Simulations for different population sizes are shown, together with expected values for both the coalescent and Wright-Fisher with large sample size. (*right*) Small samples taken from a larger simulated population agree with the coalescent expected values.

6.4 Response to selection

To validate our implementation of selection on quantitative traits, we simulated a quantitative trait with 10 QTLs with identical additive effect sizes and initial allele frequencies, which gives an approximately normal distribution of trait values in the population. We also used the `forqs` fitness function `FitnessFunction_TruncationSelection`, which selects a specified proportion of individuals at the upper tail of the trait value distribution to produce offspring for the next generation.

The Breeder's Equation from quantitative genetics (Gillespie 2004; Falconer and Mackay 1996) predicts the response to selection R from the heritability of the trait h^2 and the selection differential S:

$$R = h^2 S$$

where the S and R are the selected parent mean and offspring mean, respectively, measured as deviations from the population mean.

Simulations run with various values for the heritability and proportion of individuals selected agree with the response values predicted from the Breeder's Equation (Figure 7).

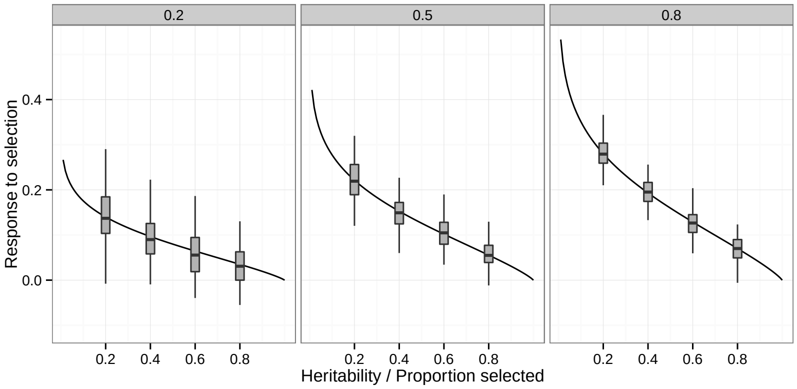

Figure 7: **Response to selection** 3 panels show simulations run at different heritability levels (.2, .5, .8), with varying proportions of selected individuals (.2, .4, .6, .8) (100 simulations each). The dark curves show the response values predicted from the Breeder's Equation.

6.5 Performance

The representation of chromosomes as haplotype chunks in `forqs` makes very efficient use of memory, independent of the size of the chromosomes. This allows, for example, simulation of entire genomes of individuals. One drawback to this approach is that memory usage grows linearly with the number of generations, due to recombination.

On a typical laptop computer, for a population size of 1 million, simulations take ∼1.5 seconds per generation for neutral scenarios and ∼3 seconds per generation with quantitative traits and selection. Decreasing the population size allows the simulation of a greater number of generations in a reasonable amount of time: for a population size of 10,000, it takes ∼3 seconds per 100 generations (without selection, with a slight increase with selection). Configuration files for these benchmark scenarios are included in the `forqs` packages (`examples/benchmark_*.txt`).

To illustrate the simulation of entire genomes, the configuration file `examples/example_qtl_random_full_human_genome.txt` specifies a scenario where two populations (each size 10,000) are selected for different optimum values of a quantitative trait (Figure 8). In this simulation, individuals have human-sized genomes (23 chromosome pairs, chromosome length 100 million base pairs), with 50 QTLs placed randomly across the genome; the simulation takes ∼2 seconds per generation (averaged over 50 generations).

Such a simulation is not feasible for forward-in-time simulators that store individual mutations in an array. For example, suppose that each locus for each individual chromosome is encoded in a single bit. Then to simulate 20,000 individuals as in the above scenario, 16GB of memory (available on high-end

computers) will permit the simulation of only 3.2 million base pairs of sequence. This is an order of magnitude smaller than the smallest human chromosome (or roughly 0.1% of the human genome).

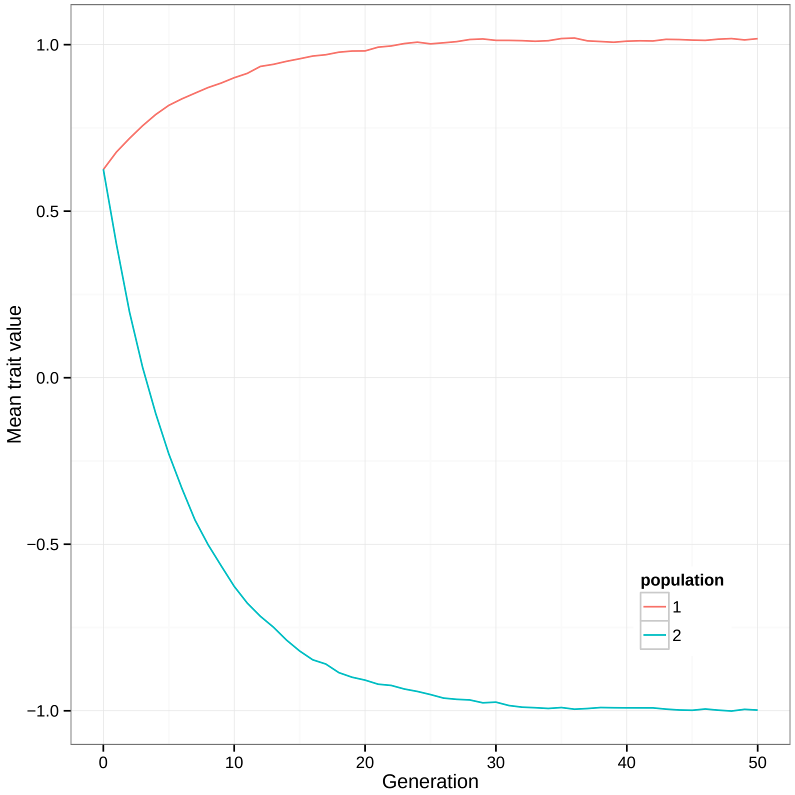

Figure 8: **Selection for different optimal values of a quantitative trait** Shown are mean trait values for two populations (each size 10,000) that are selected for different optimal trait values. In this scenario, the quantitative trait has 50 QTLs distributed randomly over 23 chromosomes, with effect sizes drawn from an exponential distribution.

7 Software development

7.1 Building the program

`forqs` is written in C++, and makes extensive use of the Boost libraries, including the Boost build system. It is regularly built and tested on OSX and Linux. In addition, Windows binaries are built using cross-compilation with gcc on Linux.

The code can be obtained via git from bitbucket:

```
git clone https://bitbucket.org/dkessner/forqs.git
```

If you have the both the Boost libraries and the Boost build system installed, you can build `forqs` with the `bjam` tool, which is the Boost build system's equivalent of `make`:

```
cd forqs/src
bjam
```

7.2 Structure of the codebase

Most code units in the `forqs` project consist of three files:

- interface (header file)
- implementation (cpp file)
- unit test (cpp file)

For example, the code for the `Locus` module can be found in the files `Locus.hpp`, `Locus.cpp`, `LocusTest.cpp`.

The unit test for each code unit is run during the build; the build is successful only if all unit tests pass.

In addition to the unit tests, there are also regression tests, consisting of scripts in the `regression_test` directory. These tests prevent unintended behavior changes in the software during new development. Most of the tests are based on the example configuration files in the `examples` directory, and consist of running `forqs` and performing a `diff` comparison with known output. The regression tests are controlled by a Makefile, and run by `make`.

The codebase includes all documentation, including the Latex source code for this document. The `forqs` Module Reference is generated by Doxygen from the source code – each module is documented using Doxygen markup in the module's header file, immediately preceding the module's class declaration.

7.3 Software architecture

This section describes some of the design details of `forqs` that may be of interest to programmers who would like to add new features.

7.3.1 Low-level data structures

These are the basic low-level data structures used by `forqs`:

- `HaplotypeChunk`: a pair of integers (*position*, *id*)
- `Chromosome`: an array of `HaplotypeChunks`
- `Organism`: an array of pairs of `Chromosomes`
- `Population`: an array `Organisms`

In this setup, `Population` can be seen as an array of arrays, which requires a significant number of memory allocations. To avoid these memory allocations, `Population` was re-implemented as a two dimensional array of `Chromosome` pairs, resulting in a roughly 30% speedup.

The implementations produce identical results, and can be used interchangeably. To accomplish this, `Population` is actually an interface class, with two concrete implementations: `Population_Organisms` and `Population_ChromosomePairs`. While the latter is faster, the former can be more convenient for testing purposes.

In order to handle both cases in a unified manner, the class `ChromosomePairRange` represents the begin/end of chromosome pairs belonging to a single individual, and `ChromosomePairRangeIterator` does the appropriate iteration through individuals, depending on the memory layout of the population.

7.3.2 Configurable modules

Configurable modules in `forqs` are represented by the `Configurable` abstract base class, which defines the common interface through which objects are instantiated and initialized based on parameters specified by the user. The top-level module (`SimulatorConfig`), all primary modules (e.g. `PopulationConfigGenerator`, `Reporter`, etc.), and all building block modules (e.g. `Trajectory`, `Locus`) implement the `Configurable` interface.

The `Configurable` interface describes the functionality required to serialize an object's configuration, which must be representable as a list of name-value pairs. (This is not actually very restrictive, since the value can be anything representable as a string, including an array of numbers).

`Configurable` objects are instantiated by `SimulationBuilder_Generic`, which handles the mapping from the module name to the actual C++ class. Each object in the user-specified configuration file is instantiated and registered by name so that other objects can obtain references to it if necessary. `SimulationBuilder_Generic` produces the final `SimulatorConfig` used by `forqs` for the simulation. (Note on the name: there were other `SimulationBuilder` classes that constructed `SimulatorConfigs` based on a more limited set of parameters – these have since been deprecated in favor of the more generic specification via configuration files.)

After instantiation, `Configurable` objects are configured/initialized in two steps. First, the `configure()` method sets any parameters specific to this module that are specified by the user. Second, after all objects have been configured, the `initialize()` method allows the modules to communicate with each other. For example, objects that need information about the lengths of the chromosomes (e.g. for recombination) can query the `PopulationConfigGenerator`, which has this information.

`Configurable` objects are instantiated, configured, and initialized in the order they are specified in the configuration file. It is important to note that while objects can obtain references to previously instantiated objects during the `configure()` step, they should not try to communicate via these references until the `initialize()` step, since the objects they refer to may not be fully initialized until then.

The main `Simulator` object interacts with `Configurable` objects through intermediate interfaces. This allows the main simulation logic to be separated from the specific behavior implemented by the various `Configurable` modules. For example, `Reporter_AlleleFrequencies` and `Reporter_TraitValues` both implement the `Reporter` interface. `Simulator` will give each `Reporter` information about the current populations, but doesn't need to know what they do with this information. As another example, the `Simulator` gives a `QuantitativeTrait` object information about individuals' genotypes and expects to receive trait values for each individual, but doesn't need to know details about how this is accomplished.

The `Configurable` interface also facilitates some features that make `forqs` more user-friendly, because it provides a translation layer between the user and program internals. For example, while 0-based indexing is used internally, from the user's perspective chromosomes and populations are numbered starting with 1. Also, `Configurable` modules can support multiple alternate parametrizations. For example, linear trajectories may be parametrized by slope-intercept or by two endpoints, and exponential distributions may be specified by the mean or the rate, depending on which is more natural for a particular scenario.

7.3.3 Mutation handling

Because `forqs` tracks haplotype chunks rather than sequences of variants, mutation is necessarily more complicated than recombination. This is because a new point mutation essentially creates a new haplotype that is identical to the original except at the mutated site. In order to accomplish this, the `VariantIndicator` must be able to be updated with the new haplotype and variant value. In addition, the haplotype's ancestry must be stored, since that haplotype chunk may contain other variants known by the `VariantIndicator`. This is implemented with the special `VariantIndicator_Mutable` that is not specified by the user. Instead, it is a wrapper class that is automatically instantiated when the user specifies a `MutationGenerator`. This wrapper class provides the functionality for updating variants, but passes calls through to the user-specified `VariantIndicator` for non-mutated loci.

7.4 Appendix: Boost libraries

The Boost C++ Libraries are an essential extension to the C++ Standard Library. In addition to providing functionality missing from the Standard Library, the Boost `filesystem` library and build system insulate the programmer from many platform-specific details.

If you have administrative access to your computer, you can use a package manager to install the Boost libraries and build system. If not, you can install Boost locally in your home directory. The following are instructions for doing this on a Unix-like system (e.g. OSX or Linux):

```
# download latest Boost package, uncompress

# install boost libraries

cd boost_???                        # go into the uncompressed directory
bootstrap.sh --help                 # see options
bootstrap.sh --prefix=$HOME/local   # install in ~/local
./b2                                # builds libraries -- get coffee (this can take a while)
./b2 install                        # installs stuff in ~/local/include/boost and ~/local/lib

# install boost build

cd tools/build/v2
./bootstrap.sh
./b2 install --prefix=$HOME/local   # puts bjam in ~/local/bin, boost-build in ~/local/share

# also:
#   put ~/local/bin in your path (to find bjam)
# you might need this in your environment:
#   export BOOST_BUILD_PATH=$HOME/local/share/share/boost-build

# to avoid bjam warning: No toolsets are configured.
# create the file user-config.jam in either ~ (home) or ~/local/share/boost-build
# with the following line (note the space before ; is necessary):
    using gcc ;
```

7.5 Appendix: Cross-compilation targeting Windows

Cross-compilation of Windows binaries from Linux can be done using tools from the `mingw-w64` project (`http://mingw-w64.sourceforge.net/`). On Debian-based systems (e.g. Ubuntu, Mint), installing package `g++-mingw-w64` will install the necessary tools and dependencies.

```
# add this line to user-config.jam, which defines the toolset gcc-windows
# (you need to tell Boost build which g++, ar and ranlib to use):
using gcc : windows : i686-w64-mingw32-g++ : <archiver>i686-w64-mingw32-ar
    <ranlib>i686-w64-mingw32-ranlib ;

#   build the Boost libraries (or at least filesystem and system) for Windows
#   (you may need to fiddle with these command line parameters -- these worked for me),
#   from your Boost source dir:
bjam toolset=gcc-windows --prefix=$HOME/local_win32 threading=multi
```

```
  target-os=windows link=static threadapi=win32 --without-mpi
  runtime-link=static --without-python -sNO_BZIP2=1 --layout=tagged

# note: LIBRARY_PATH doesn't appear to work for cross-compilation, but you can
# put the Boost library files directly in /usr/i686-w64-mingw32, where the various
# development headers/libs are installed for mingw-w64

# the forqs Jamroot contains a target ``windows'', which will build Windows binaries
# and put them in forqs/bin_windows:
bjam windows
```

References

Falconer, D. S. and Mackay, T. F. (1996). *Introduction to Quantitative Genetics (4th Edition)*. Benjamin Cummings, 4 edition.

Fu, Y. X. (1995). Statistical properties of segregating sites. *Theor Popul Biol*, **48**(2), 172–197.

Gillespie, J. H. (2004). *Population Genetics: A Concise Guide*. Johns Hopkins University Press, 2nd edition.

Wakeley, J. and Takahashi, T. (2003). Gene genealogies when the sample size exceeds the effective size of the population. *Mol. Biol. Evol.*, **20**(2), 208–213.
